# Development of epithelial-mesenchymal transition-related lncRNA signature for predicting survival and immune microenvironment in pancreatic cancerwithexperiment validation

**DOI:** 10.1080/21655979.2021.2000197

**Published:** 2021-12-02

**Authors:** Yong Gao, Jinhui Liu, Baobao Cai, Qun Chen, Guangfu Wang, Zipeng Lu, Kuirong Jiang, Yi Miao

**Affiliations:** a Pancreas Center, the First Affiliated Hospital of Nanjing Medical University, Nanjing, People’s Republic of China; b Department of Gynecology, The First Affiliated Hospital of Nanjing Medical University, Nanjing, People’s Republic of China; c Pancreas Center, the Affiliated BenQ Hospital of Nanjing Medical University, Nanjing, People’s Republic of China

**Keywords:** Pancreatic cancer, prognostic signature, lncRNA, tumorenvironment, epithelial–mesenchymal transition

## Abstract

Long non-coding RNAs (LncRNAs) have crucial function in epithelial–mesenchymal transition (EMT) in pancreatic cancer. It is necessary to comprehensively analyze the potential role of EMT-related lncRNA in pancreatic cancer. In the present study, genomic data of pancreatic cancer from the TCGA database were downloaded and we found 368 EMT-related lncRNAs. According to the expression characteristics of prognostic-related lncRNAs, all samples could be divided into two clusters with different clinical outcomes and different tumor microenvironments. Moreover, an eleven EMT-related lncRNAs signature was established and verified. Patients with pancreatic cancer in the high-risk group had a shorter overall survival than those in the low-risk group and the signature could act as an independent prognostic factor. Further analysis suggested that the EMT-related lncRNAs might affect the prognosis of patients through immune mechanisms. All findings indicated that the signature and eleven lncRNAs might serve as potential prognostic biomarkers and therapeutic targets in the treatment of pancreatic cancer.

## Introduction

As one of the most deadly human malignancy, pancreatic cancer ranks fourth on the list of cancer-related causes of death in the US [1]. The prognosis of patients suffering from pancreatic cancer remains poor, as more than half patients have reached the metastatic stage before being diagnosed, despite advances in surgical innovations, chemotherapy and immunotherapy [2]. Hence, it is still of great clinical significance to explore effective and promising biomarkers and therapeutic targets for the patients with poor prognosis.

Epithelial–mesenchymal transition (EMT) is a biological process by which epithelial cells are transformed into cells with a mesenchymal phenotype by a specific program [3]. EMT not only plays an important role in the tumorigenesis of pancreatic cancer, but also affects the progression of pancreatic cancer [4]. The progressive development of tumor cells into a mesenchymal state makes the tumor more susceptible to have metastasis, and it could also be a reason for the resistance of tumors to radiotherapy and chemotherapy [5,6]. In addition, several studies have shown the component of tumor microenvironment, such as immune cells, cancer-associated fibroblasts and extracellular matrix, drive and sustain EMT in pancreatic cancer [7,8]. A better understanding of the properties of EMT could help develop merging and effective therapies. Long non-coding RNAs, abbreviated as lncRNAs, are non-coding RNA with more than 200 nucleotides in length. LncRNAs can be involved in oncogene and tumor suppressor genes network regulation in the epigenetic level, transcriptional level or post-transcriptional level. In pancreatic cancer, various lncRNAs have been found to act important role in tumor progression via diverse biological mechanisms including tumor growth, metastasis and drug resistance [9]. Recently, LncRNAs have shown crucial function in EMT in pancreatic cancer [10–13]. However, existing studies only explored the single role of individual lncRNAs related to EMT in pancreatic cancer and integrated analysis on lncRNAs related to EMT are rarely carried out. Consequently, it is necessary to comprehensively analyze the potential role of EMT-related lncRNA in pancreatic cancer.

In the current research, by analyzing the transcriptome expression data from the TCGA database, the potential lncRNAs associated with EMT were investigated and extracted. All pancreatic cancer samples were stratified into two clusters based on EMT-associated lncRNAs. Subsequently, a novel signature was constructed with eleven EMT-related lncRNA for accurately predicting overall survival of patients with pancreatic cancer. In addition, diverse immune status and tumor environment were observed among pancreatic cancer samples from different groups. The signature and eleven lncRNAs in this work might serve as potential prognostic biomarkers and therapeutic targets in the treatment of pancreatic cancer.

## Methods

### Data preparation and processing

The expression profile of all genes from 178 pancreatic cancer samples and 4 normal tissues were obtained from The Cancer Genome Atlas (TCGA) database on 28 February 2021. Clinical information corresponding to the samples were also extracted. Guided by gene annotation in the GENCODE project [14], the mRNA and lncRNA expressions were extracted into a matrix separately.

## Identification of lncRNA related to epithelial-mesenchymal transition

Genes related to the EMT was obtained from the dbEMT database, a comprehensive gene resource for EMT and mRNA expression data was extracted. Then, Pearson correlation analysis was used to identify a list of lncRNAs related to EMT, with correlation coefficient |r| > 0.4 and p < 0.001 as the threshold. Subsequently, univariable Cox regression analysis was performed to screen EMT-related lncRNAs predicting overall survival with p < 0.001 as the threshold and expression of these lncRNAs between normal and tumor samples was shown with R Package pheatmap and ggpubr.

## Consensus clustering

R package Consensus Cluster Plus was used to perform consensus clustering. The cumulative distribution function (CDF) and consensus matrix were used to evaluate the optimal number of subgroups. The overall survival between different clusters was compared by the Kaplan-Meier method with log-rank test. In order to understand the correlation between different clusters distinguished through the expression of EMT-related lncRNAs and immune features in pancreatic cancer. Stromal Score, immune Score, and ESTIMATE Score based on transcriptome profiles of pancreatic cancer were calculated with the support of ESTIMATE algorithm. Immune cell infiltration was accurately assessed by the CIBERSORT deconvolution algorithm (http://cibersort.stanford.edu/) [15] as well, and subsequently the difference between two cluster was further confirmed.

## Construction and validation of prognostic signatures

Tumor samples were randomly classified into the training cohort to build predictive signature and the testing cohort, together with the entire cohort, for signature verification (n = 52). All EMT-related lncRNAs predicting overall survival were assembled into lasso regression model to build a prognostic risk model. The risk score was accumulated using the regression coefficients (β) from the lasso regression model to weigh the weight of the selected lncRNAs. Risk score = βlncRNA(1) × expression of lncRNAs(1) + βlncRNA(2) × expression of lncRNAs(2) + … +βlncRNA(n) × expression of lncRNAs(n). Patients with scores above the median risk score were placed in the high-risk group, while those with scores below the median risk score were assigned to the low-risk group. Kaplan-Meier method with log-rank test was used to compere the overall survival of patients in low-risk and high-risk groups. Receiver operating characteristic (ROC) curve was plotted to assess the predictive performance of the prognostic risk model in comparison with clinicopathological feature. The area under the ROC curve (AUC) was subsequently calculated. Univariate and multivariate Cox regression analyses were used to determine whether the prognostic risk model or other clinicopathological characteristics was an independent risk factor.

## Gene set variation analysis (GSVA) analysis

In order to the differences of enriched gene sets between different groups, GSVA analysis was performed between different clusters, and different risk groups with R package GSVA and limma.

## Evaluation of correlation with immune tumor microenvironment

CIBERSORT algorithm, an analytical tool to provide an estimation of the abundances of member cell types in a mixed cell population using gene expression data, was applied to retrieve the abundance of tumor-infiltrating immune cell in each sample. Subsequently, the tumor microenvironment in different groups was analyzed and correlation between all types of immune cells and risk score was calculated.

## Quantitative RT-PCR (qRT-PCR)

All patients’ samples were collected from Pancreas Center, The First Affiliated Hospital of Nanjing Medical University. We analyzed 10 pancreatic cancer tissues and paired normal tissues. Total RNA was extracted from samples using the Total RNA Kit according to the manufacturer’s instructions. After spectrophotometric quantification, 1 μg of total RNA in a final volume of 20 μl was then reverse transcribed with PrimeScript RT Master Mix according to the manufacturer’s instructions: 37°C for 15 minutes, 85°C for 5 seconds and 4°C before storage. Then qRT-PCR was performed using SYBR green reagent (A25742, Life Technologies) on a Step One Plus Real-Time PCR system according to the manufacturer’s protocol: a total volume of 10 μl containing 1 μl template, 0.1 μl 5 μM forward/reverse primer and 5 μl PCR Master Mix, 95°C for 10 minutes, 40 cycles of denaturation at 95°C for 15 seconds and extension at 60°C for 1 minute.18S rRNA was used as an internal control. The 2− ΔΔCt method was used to analyze the relative expression of target genes. Primers sequences are designed and synthesized by Sangon Biotech (Shanghai, China). The lncRNA sequences were put into the software Primer3Plus, and the primers with the highest score were selected after specificity confirmation trough NCBI blast. The primer sequences used were listed as follows:

**Table:** 

	Forward Primer(5ʹ-3ʹ)	Reverse Prime(5ʹ-3ʹ)
CASC8	GTCCTCAGCGGAAACAGC	GGCCTCCACATCAAGTCATA
SH3PXD2A-AS1	TCCCACAGAATACCAGAA	TGACTCAAAGTAGAGGTGC
TRAF3IP2-AS1	TTTACCGCAACGACAACT	AAGGACCAAACGAGATGA
PAN3-AS1	GCAGCACCTGTTATTCTC	TCTAAGCAGTGTTGTCCC
LINC01133	GGGAATGGTTGGGAGGAG	TGGGCTCAAGGAATCTGAATAG
18S rRNA	CGAACGTCTGCCCTATCAACTT	ACCCGTGGTCACCATGGTA

## Statistical analysis

R version 3.6.3 and R package (survival, limma, pheatmap, reshape2, ggpubr, ConsensusClusterPlus, survminer, ggplot2, corrplot, estimate, vioplot, timeROC, ggExtra) were applied for all statistical analyses. Student’s t-test was used to compare the difference between two groups. P ≤ 0.05 was considered statistically significant if not specified.

## Results

In the present study, we extracted and analyzed transcriptome expression data from TCGA database to study lncRNAs associated with EMT. According to EMT-realated lncRNAs, all pancreatic cancer samples could be divided into two clusters. Subsequently, we constructed a novel signature with 11 EMT-associated lncRNAs to predict overall survival of patients with pancreatic cancer in training, testing and entire sets. Furthermore, there were differences in immune status and tumor environment in different groups of pancreatic cancer samples.

## Overview of lncRNA related to EMT

**Figure 1.**
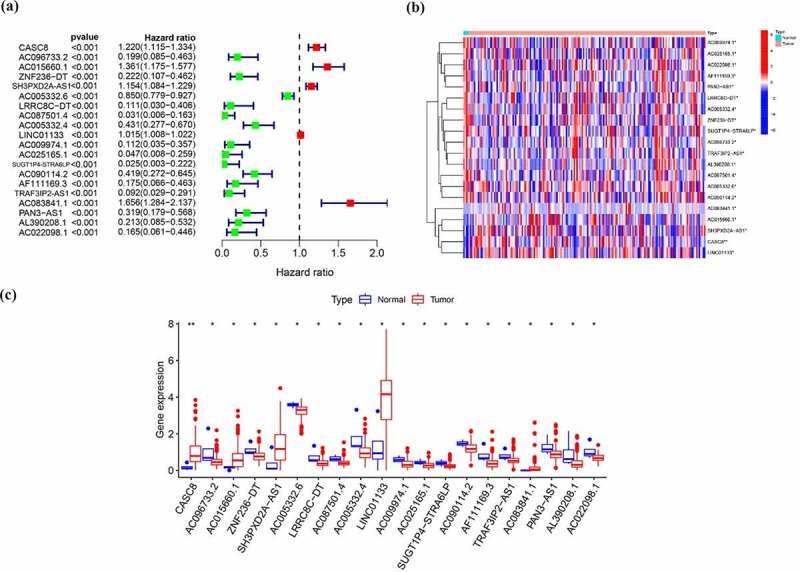
EMT-related lncRNAs in pancreatic cancer. (a) Univariable Cox regression analysis of EMT-related lncRNAs (b, c) Expression of EMT -related lncRNAs predicting overall survival between tumor and normal samples

In total, 1184 EMT-related genes were retrieved from the EMT gene database (Supplementary Table 1). Then, 368 lncRNAs have correlation with above genes in expression based on Pearson correlation with |r| > 0.4, P < 0.001 as threshold were regarded as EMT-related lncRNAs (Supplementary Table 2). Univariable Cox regression analysis was used to assess the relationship between lncRNAs related to EMT and overall survival. Among them, 20 lncRNAs were determined as prognosis-related lncRNA, including 5 lncRNAs associated with poor survival and 15 lncRNAs related to good prognosis (Table 1, Figure 1(a)). In addition, these 20 EMT-related lncRNAs predicting overall survival were all differentially expressed between tumor and normal samples as shown in Figure 1(b,c).

**Table 1.** Univariable Cox regression analysis of EMT-related lncRNA in patients with pancreatic cancer

lncRNA	HR	HR.95 L	HR.95 H	pvalue
SUGT1P4-STRA6LP	0.024684	0.002746	0.221856	0.000953
AC087501.4	0.030751	0.005801	0.163022	4.29E-05
AC025165.1	0.0468	0.008461	0.258854	0.00045
TRAF3IP2-AS1	0.09232	0.029328	0.290608	4.66E-05
LRRC8C-DT	0.111277	0.030471	0.406367	0.000892
AC009974.1	0.112476	0.035427	0.357091	0.00021
AC022098.1	0.16519	0.061213	0.445784	0.000378
AF111169.3	0.174814	0.066043	0.462727	0.000445
AC096733.2	0.198703	0.085326	0.462735	0.000179
AL390208.1	0.213203	0.085437	0.532034	0.000925
ZNF236-DT	0.222214	0.10683	0.462219	5.69E-05
PAN3-AS1	0.318505	0.178578	0.568076	0.000106
AC090114.2	0.418899	0.271935	0.645288	7.91E-05
AC005332.4	0.430604	0.276584	0.670392	0.000191
AC005332.6	0.850105	0.779314	0.927327	0.000251
LINC01133	1.015099	1.007798	1.022452	4.71E-05
SH3PXD2A-AS1	1.154071	1.083836	1.228856	7.71E-06
CASC8	1.21957	1.115113	1.333812	1.39E-05
AC015660.1	1.361011	1.174653	1.576935	4.09E-05
AC083841.1	1.656427	1.283987	2.136899	0.000103

## Stratification of pancreatic cancer based on the LncRNA related to EMT

**Figure 2.**
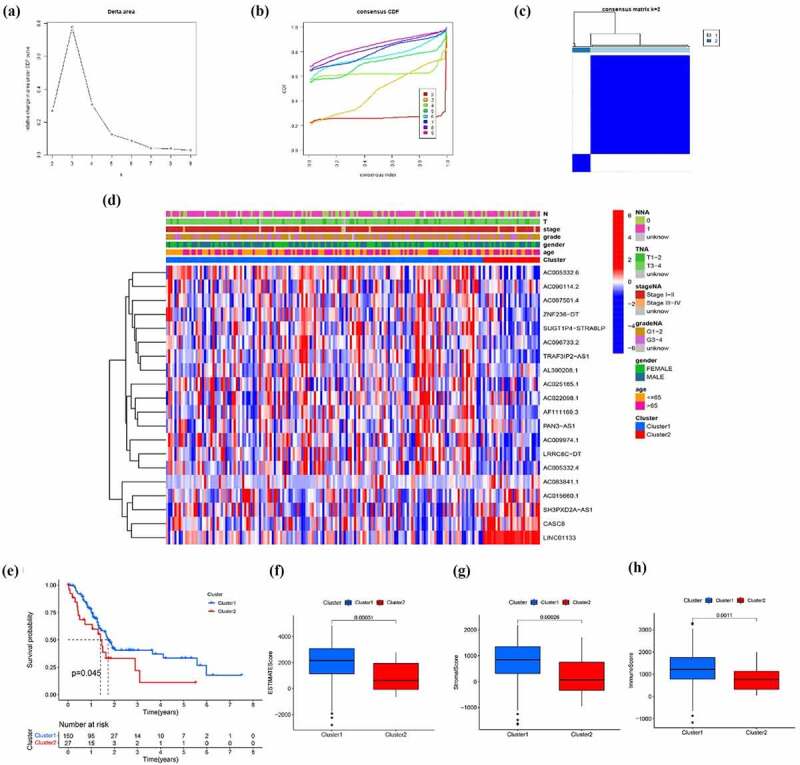
Clusters distinguished by the expression of EMT-related lncRNA. (a) Relative change in the area under the CDF curve for k = 2 to k = 9 (b) Consensus clustering CDF for k = 2 to k = 9 (c) Consensus clustering matrix of samples from the TCGA dataset for k = 2 (d) Expression of EMT-related lncRNAs and distribution of main clinicopathological characteristic in two clusters (e) Survival analysis of patients in two clusters (f-h) ESTIMATE score, immune score and stromal score of cluster 1 and cluster 2

**Figure 3.**
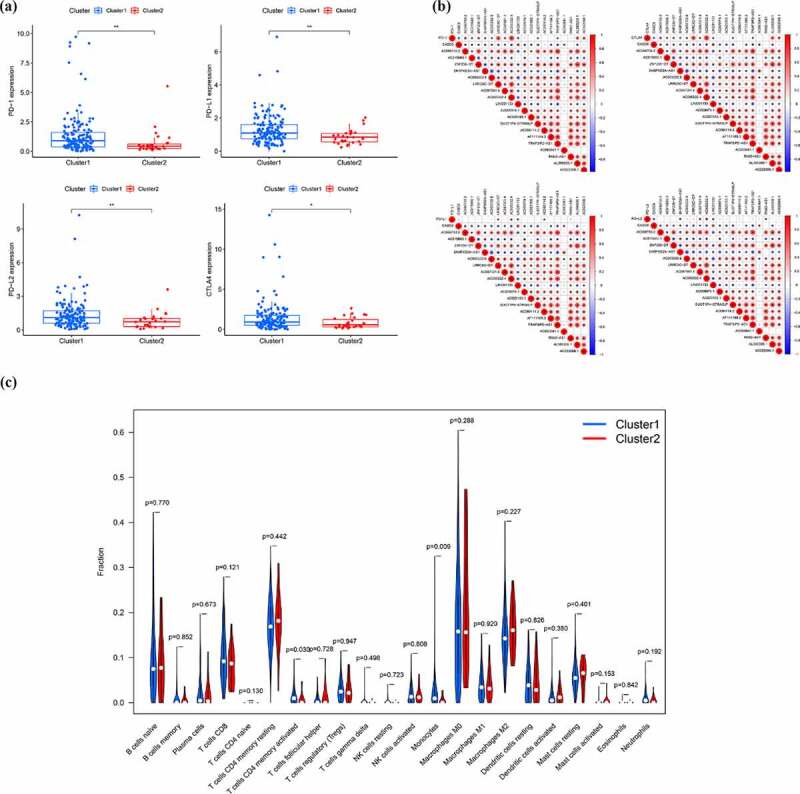
EMT-related lncRNA clusters significantly associated with the immune microenvironment. (a) Expression level of four immune checkpoints in two clusters (b) Association between PD-1 and EMT-related lncRNA (c) Immune cells infiltration in two clusters (d) Differentially infiltrating immune cells in two clusters

In order to preliminarily understand the underlying mechanism of the biological function of EMT-related lncRNAs, the unsupervised clustering analysis was performed. The cluster stability increased between k = 2 and k = 9 (Figure 2(a,b)). The consensus matrix heatmap suggested that samples could be reasonably divided into two clusters (Figure 2(c)). Subsequently, the clinicopathological and immune features of different EMT-related lncRNA clusters were analyzed. As illustrated in Figure 2(d), there was no significant difference in distribution of main clinicopathological characteristic between two groups. The results of K-M analyses indicated that the prognosis of patients in cluster 1 is better than that in cluster 2 (Figure 2(e)). At the same time, the ESTIMATE score, immune score and stromal score of cluster 1 were significantly higher than that of cluster 2, which means that the samples in cluster 1 have a lower purity of the tumor component (Figure 2(f,g,h)). To further explore tumor environment, the expression levels of several immune checkpoints such as PD-1, PD-L1, PD-L2, and CTLA-4 were compared. It was found that the expression levels of the four immune checkpoints in the cluster 1 were all obviously higher than that in the cluster 2 (Figure 3(a)). The expression of PD-1, PD-L1, PD-L2, and CTLA-4 are associated to most EMT-related lncRNA (Figure 3(b)). Besides, by comparing the composition of 22 immune cells, the expression level of T cells CD4 memory activated and Monocytes in cluster 1 is significantly higher than that in cluster 2 (Figure 3(c)). Moreover, GSVA analysis was performed to compare differences of enriched gene sets between cluster 1 and cluster 2 (Supplementary Figure 1). Overall, the above results indicated that the subgroups of pancreatic cancer samples with different immune infiltration characteristics could be distinctly distinguished by consensus cluster based on EMT-related lncRNAs.

## Construction and validation of the EMT-related 11-lncRNA signature

**Figure 4.**
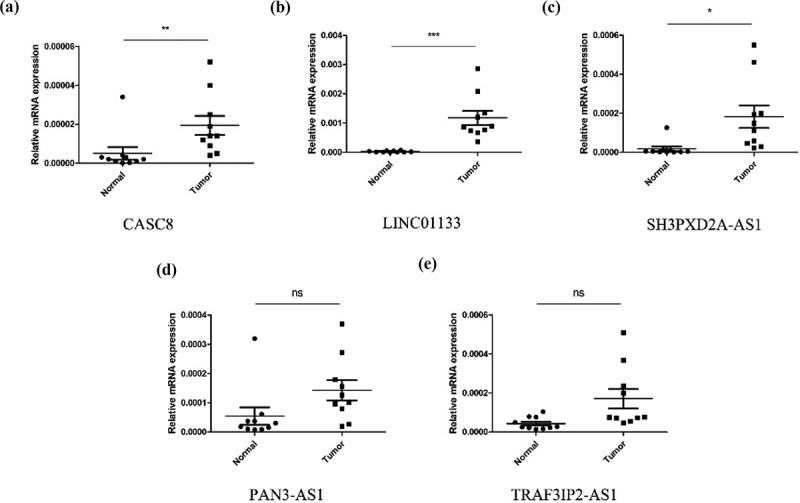
The expression level of CASC8 (a), LINC01133 (b), SH3PXD2A-AS1 (c), PAN3-AS1 (d) and TRAF3IP2-AS1 (e) mRNA in cancerous and normal tissues from our samples

**Figure 5.**
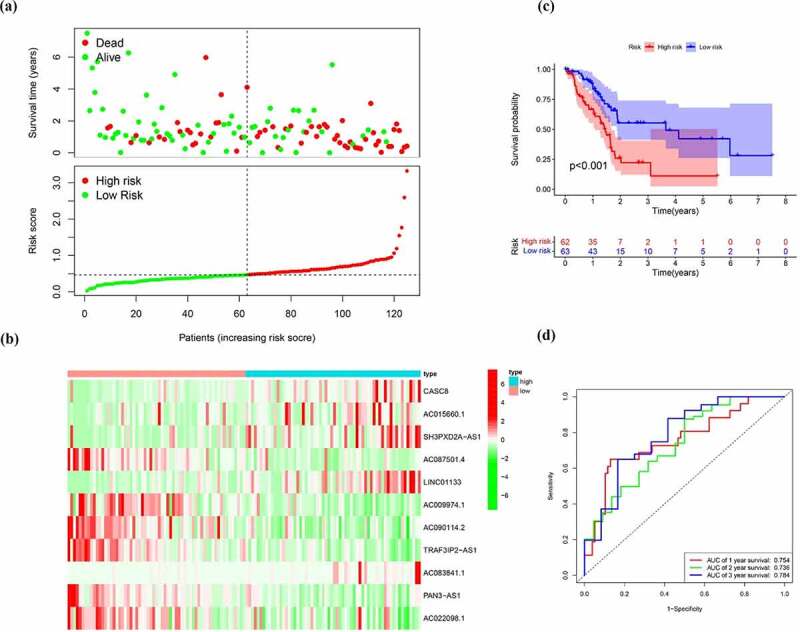
Identification of the EMT-related lncRNA signature in the training cohort. (a)Survival status and risk score distribution in the high- and low-risk groups. Green dots represent surviving patients; red dots represent dead patients (b) Kaplan-Meier curve analysis of overall survival in the high- and low-risk groups (c) Expression patterns of 11 EMT-related lncRNAs in high- and low-risk groups (d) ROCcurve analysis

**Figure 6.**
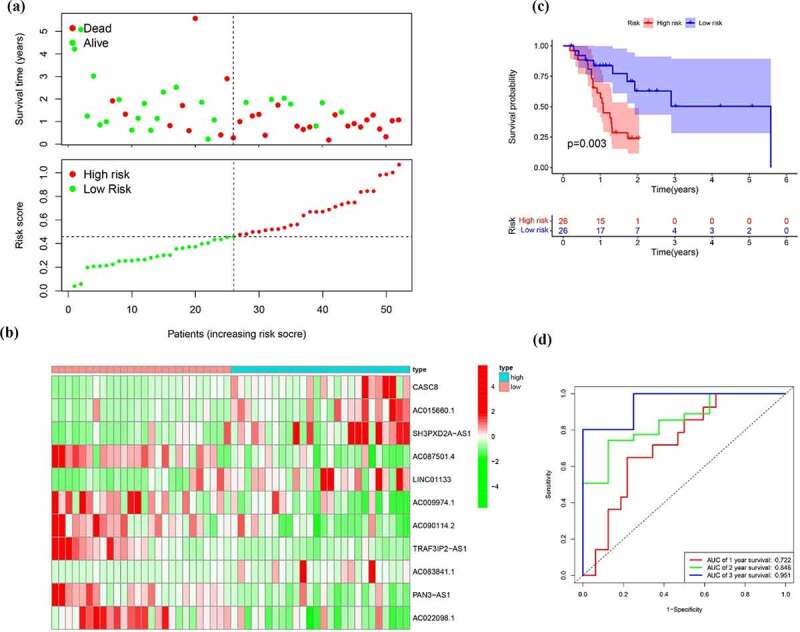
Verification of the EMT-related lncRNA signature in the test cohort. (a)Survival status and risk score distribution in the high- and low-risk groups. Green dots represent surviving patients; red dots represent dead patients (b) Kaplan-Meier curve analysis of overall survival in the high- and low-risk groups (c) Expression patterns of 11 EMT-related lncRNAs in high- and low-risk groups (d) ROC curve analysis

**Figure 7.**
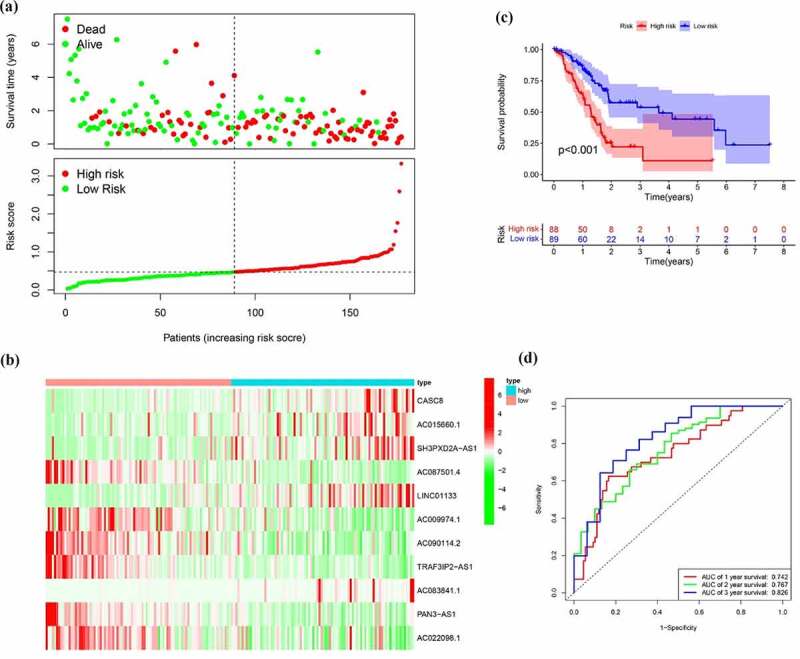
Verification of the EMT-related lncRNA signature in the entire cohort. (a)Survival status and risk score distribution in the high- and low-risk groups. Green dots represent surviving patients; red dots represent dead patients (b) Kaplan-Meier curve analysis of overall survival in the high- and low-risk groups (c) Expression patterns of 11 EMT-related lncRNAs in high- and low-risk groups (d) ROC curve analysis

To build a prognostic signature, the LASSO Cox regression model was used to narrow the most robust lncRNAs for prognosis. Ten-fold cross-validation was applied to overcome the over-fitting. As a result, a prognostic signature containing 11 EMT-related lncRNA was established. The coefficients of each lncRNA included in the signature were shown in Table 2. According to the expression level of 11 EMT-related lncRNAs and corresponding coefficients, each pancreatic cancer sample was given a risk score. Risk score = 0.057 × expression of CASC8 + 0.030 × expression of AC015660.1 + 0.021 × SH3PXD2A-AS1 – 0.161 × AC087501.4 + 0.006× LINC01133-0.235 × expression of AC009974.1–0.317 × expression of AC090114.2–0.300 × TRAF3IP2-AS1 + 0.221 × AC083841.1–0.328× PAN3-AS1- 0.162× AC022098.1. In our samples, the expression of CASC8, LINC01133 and SH3PXD2A-AS1 was higher in tumor tissues than that in paired normal tissues (Figure 4(a-c)). On the contrary, there is no significant difference in the expression of PAN3-AS1 and TRAF3IP2-AS1 between tumor and normal tissues (Figure 4(d,e)). According to the median level of risk scores, all pancreatic cancer samples were divided into high-risk group with high scores and low-risk group with low scores. The distribution of risk score, survival status, and expression of 11 hub lncRNAs in the training set were demonstrated in Figure 5(a-b). Kaplan-Meier analysis was performed to assess the effect of risk score on survival and the result showed a remarkable difference in overall survival between high-risk and low-risk groups (P < 0.001, Figure 5(c)). In addition, the 1-, 2- and 3-year AUCs in the training set were 0.754, 0.736, and 0.784, respectively (Figure 5(d)). To confirm the stability of the model, the testing cohort and entire cohort were used to further test the prognostic effects of the risk model. The distributions of risk score, survival status, and expression of 11 hub lncRNAs in the testing and entire sets were shown in Figure 6(a,b) and Figure 7(a,b). Similar to the training set, the overall survival of patients in the high-risk group was remarkably shorter than that of patients in the low-risk group in both the testing (P = 0.003) and entire sets (P < 0.001) (Figures 6(c), 7(c)). Besides, the results of ROC analysis verified the established signature and had satisfactory predictive accuracy in the test cohort and the entire cohort (Figures 6(d), 7(d)). Moreover, there is difference of enriched gene sets between high-risk and low-risk groups using GSVA analysis. (Supplementary Figure 2).

**Table 2.** The coefficients of EMT-related lncRNA in the signature

lncRNA	Coef
CASC8	0.05715
AC015660.1	0.030047
SH3PXD2A-AS1	0.021271
AC087501.4	−0.16136
LINC01133	0.005699
AC009974.1	−0.23477
AC090114.2	−0.31673
TRAF3IP2-AS1	−0.30038
AC083841.1	0.221318
PAN3-AS1	−0.32751
AC022098.1	−0.1625

## Prognostic value and clinical utility of EMT-related 11-lncRNA signature

**Figure 8.**
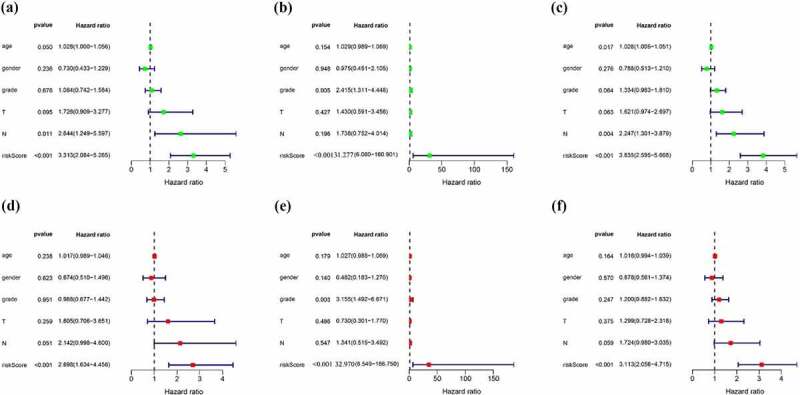
Cox regression analysis evaluating independent prognostic value of the risk score. Univariate Cox regression analysis of age, gender, grade, T stage, N stage and risk score in the (a) training cohort (b) test cohort (c) entire cohort. Multivariate Cox regression analysis of age, gender, grade, T stage, N stage and risk score in the (d) training cohort (e) test cohort (f) entire cohort

**Figure 9.**
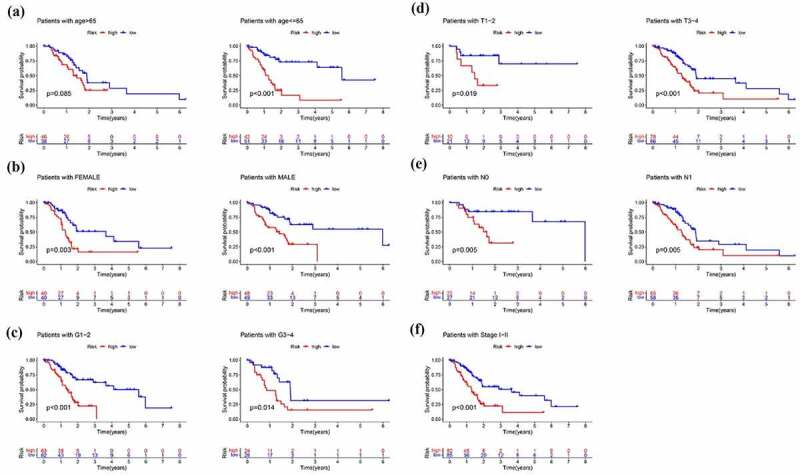
Subgroup analysis of the signature. Prognostic value of risk score in patients with different (a) ages (b) gender (c) grade(d) T stage (e) N stage (f) TMN stage

Next to the establishment of the signature, we then evaluated application value in clinical practice of risk signature in pancreatic cancer patients in the training, test and entire cohort. In order to evaluate whether the 11-lncRNA signature was an independent prognostic indicator for pancreatic cancer patients, univariate and multivariate Cox regression analyses were conducted. Univariate Cox regression analysis showed the hazard ratio (HR) of risk score and 95% CI were 3.313 (2.084–5.265) in the training set, 31.277 (6.080–160.901) in the testing set, and 3.835 (2.595–5.668) in the entire set (Figure 8(a,c,e)). It is showed in multivariate Cox regression analysis that the HR of risk score and 95% CI were 2.698 (1.634–4.456), 32.970 (6.549–186.750), and 3.113 (2.056–4.715) in training, testing, and entire set, respectively (Figure 8(b,d,f)). Furthermore, we evaluated whether the signature has the same predictive impact in subgroups from the entire set. As revealed in Figure 8, the risk score could distinguish the prognosis in patients no more than 65 years old (Figure 9(a)), male patients and female patients (Figure 9(b)), patients with G1-2 and G3-4 (Figure 9(c)), T1–2 and T3-4 (Figure 9(d)), N0 and N1 (Figure 9(e)) and Stage I–II (Figure 9(f)). In aggregate, the above results indicated that the built signature could be used as a prognostic differentiator with great promise for pancreatic cancer patients.

## Different immune status and tumor environment between high-risk and low-risk groups

**Figure 10.**
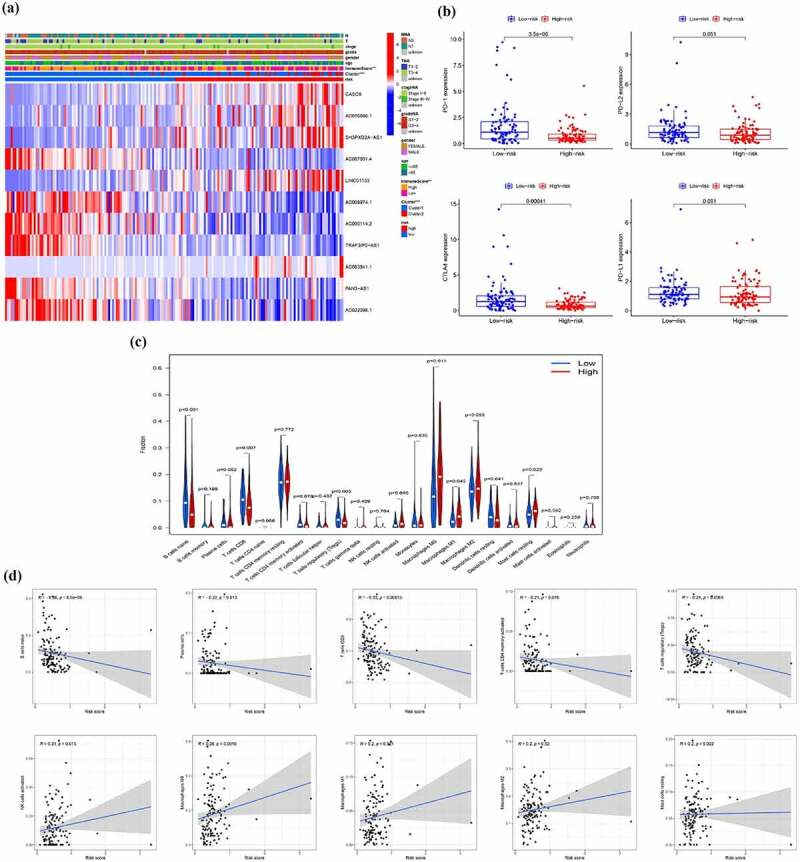
Different immune status and tumor environment between high-risk and low risk groups. (a) A heatmap showing expression of EMT-related lncRNAs and distribution of main clinicopathological characteristic in high-risk and low risk groups (b) Expression level of four immune checkpoints in high-risk and low risk groups (c) Immune cells infiltration in high-risk and low risk groups (d) Correlation between immune cells and risk score

First, we found the immune score between the high-risk and low-risk groups show the significant difference (Figure 10(a)). To further explore tumor environment, we compared the expression levels of PD-1, PD-L1, PD-L2, and CTLA-4 between high and low risk groups. Compared with the low-risk group, the expression levels of PD-1 and CTLA4 were significantly lower in the high-risk group and there was no significant difference in the expression of PD-L1 and PD-L2 between the two groups (Figure 10(b)). Besides, the differences of infiltration of all types of immune cells in high- and low-risk group (Figure 10(c)) and the correlation between all types of immune cells and risk score were calculated (Figure 10(d)). The results indicated that the risk score is negatively correlated with the infiltration of B cells naive, Plasma cells, T cells CD8, T cells CD4 memory activated and T cells regulatory (Tregs), while positively related to the infiltration of NK cells activated, Mast cells resting, Macrophages M0, M1 and M2. These results demonstrated that there were differences in the immune microenvironment between the high- and low-risk groups.

## Discussion

Pancreatic cancer is an extremely malignant tumor with unsatisfactory prognosis [16] and the number of patients with pancreatic cancer has increased year by years. Although the diagnostics and treatments are advancing, insufficient effective prognostic models or biomarkers for predicting outcomes remain an urgent problem to be solved in the management of patients with pancreatic cancer. In this present study, a prognostic model was established.

In our research, firstly, Pearson correlation analysis was employed to distinguish lncRNAs related to EMT, and 368 lncRNAs were obtained. Subsequently, according to the expression characteristics of these lncRNAs, all samples could be divided into two clusters with different clinical outcomes and different tumor microenvironments. Finally, an eleven EMT-related lncRNAs signature was established in the training cohort and verified in both validation cohort and entire cohort. Patients with pancreatic cancer in the high-risk group had a shorter overall survival than those in the low-risk group and the signature could act as an independent prognostic factor. All findings suggested that the EMT-related lncRNAs might affect the prognosis of patients through immune mechanisms and the signature could be used to predict the prognosis of patients with pancreatic cancer.

Nowadays, based on clinical features, transcriptome, proteomics and radiomics, more and more cancer prognostic model and tumor potential prognostic biomarkers are emerging. Clinicians have more weapons in their prediction of overall survival of patient with pancreatic cancer. Transcriptome data models are the most common models. Differentially expressed genes between normal and tumor tissues [17] and specific gene set, such as immune-related gene set [18] and oxidative stress-related gene set [19], were used to construct risk model to predict poor outcomes. Alizadeh *et al*. found three differentially expressed circulating miRNAs in patients, namely hsa-miR-1469, hsa-miR-663a and hsa-miR-532-5p, might have close relationship with the prognosis of patients with pancreatic cancer [20]. In addition, the ratio of common clinical blood test results could be used as an effective tool. Michael Stotz *et al*. identified the Lipase/Amylase ratio in peripheral blood as an independent prognostic factor [21]. Zhong *et al*. proposed that the baseline glucose-to-lymphocyte ratio is an independent prognostic factor for patients with pancreatic cancer [22]. Furthermore, Yosuke Iwatate *et al*. established a link between radiomics data and p53 mutations, which is associated with poor prognosis. Abnormal expression of PD-L1, could also be predicted by radiomics data, which further had aid in the development of precision medicine [23]. Eugene J Koay used a voxel-based method and machine learning-based analysis to build a predictive score model. In their model, low score group was associated with improved outcomes. The research demonstrating epigenetic aberrations were involved in the development and progression. Kong *et al*. utilized differentially methylated CpG sites of genes to construct a risk score prognostic prediction system [24]. According to our knowledge, although lncRNA plays an important role in the development of pancreatic cancer, it is rarely used to established prognostic model. In our study, we selected EMT-related lncRNAs as predictors and constructed a model that could distinguish the immune microenvironment and prognosis of pancreatic cancer. Our model has stable results in the training set and the test set, suggesting that it could be used for clinical.

The function of some lncRNAs s in our model has been studied in tumor or other disease. It has been shown that CASC8, cancer susceptibility candidate 8, has been shown to be a tumor susceptibility gene [25]. The low expression of CASC8 reduces the malignant biological behavior in non-small cell lung cancer [26]. In pancreatic cancer, CASC8 is specifically expressed at a high level and associated with poor prognosis [27], which is consistent with our results. LINC01133 was first reported in 2015 to be highly expressed in lung squamous cell carcinoma and then its role in tumors has been fully explored. LINC01133 could promote the malignant behaviors in several tumor, such as lung adenocarcinoma [28], hepatocellular carcinoma [29], triple-negative breast cancer [30] and nasopharyngeal carcinoma [31]. Similarly, LINC01133 could contribute to the tumorigenesis and progression in pancreatic cancer through numerous pathways [32,33]. On the other hand, LINC01133 could confers tumor-suppressive functions in gastric cancer [34] and ovarian cancer [35]. TRAF3IP2-AS1, expressed from TRAF3IP2, is a natural antisense lncRNA and has decreased expression in NONO-TFE3 translocation renal cell carcinoma, which promotes progression of tumor [36]. Besides, in IL-17-related autoimmune diseases, such as psoriasis and multiple sclerosis, TRAF3IP2-AS1 may represent attractive therapeutic targets [37]. Out results illustrated that TRAF3IP2-AS1 might predict a better prognosis in patient with pancreatic cancer. SH3PXD2A-AS1 was also an antisense transcript transcribed from SH3PXD2A and overexpressed in colorectal cancer. SH3PXD2A‐AS1 acted as an oncogene that could promote cellular proliferation, invasion and migration [38]. We found it was obvious that the high expression of SH3PXD2A-AS1 is related to the poor prognosis in pancreatic cancer. For other lncRNAs in the model, few mechanism researches have been performed in pancreatic cancer. These lncRNAs have also been used to predict the prognosis of other cancers [39] with the value of in-depth study.

Although our research is somewhat innovative, there still exist shortcomings in our research. First, our model is built based on public database with internal validation and its accuracy requires external data for further verification. Second, even though we provide a new approach of thinking about the data sources used to construct prognostic models, our model is not necessarily superior to all other existing models. At last, the specific mechanisms of some key genes in the model can be further explored. In the future, it is necessary to establish a better prognostic nomogram with complete clinical information and sequencing data from more centers.

## Conclusion

To sum up, we used EMT-related lncRNAs identified in pancreatic cancer to establish a 11-lncRNA signature with significant clinical value for predicting prognosis. In addition, different groups, which were distinguished based on lncRNA expression characteristics, displayed different immune statuses and had different tumor microenvironment. Therefore, this study identified several new potential targets, providing new ideas for the treatment of pancreatic cancer patients.

## Supplementary Material

Supplemental Material

## Data Availability

All data and material used or analyzed during the current study are available from the corresponding author on reasonable request.
