# Supplementary material for: Development of epithelial-mesenchymal transition-related lncRNA signature for predicting survival and immune microenvironment in pancreatic cancerwithexperiment validation
Source: Bioengineered. 2021 Dec 2;12(2):10553–67. doi: 10.1080/21655979.2021.2000197 (PMC8809919; doi:10.1080/21655979.2021.2000197)
Supplement: Supplemental Material [file KBIE_A_2000197_SM7579.zip › supplementary/Supplementary_Figure.docx]

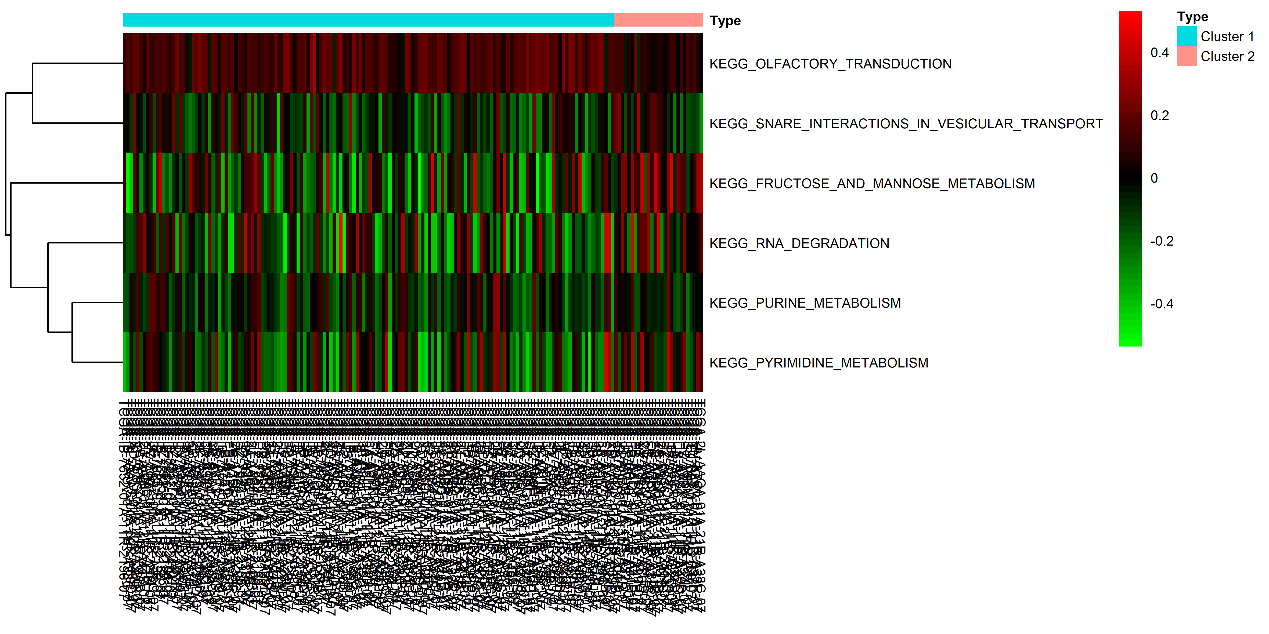


**Supplementary Figure 1** GSVA analysis between Cluster 1 and Cluster 2.


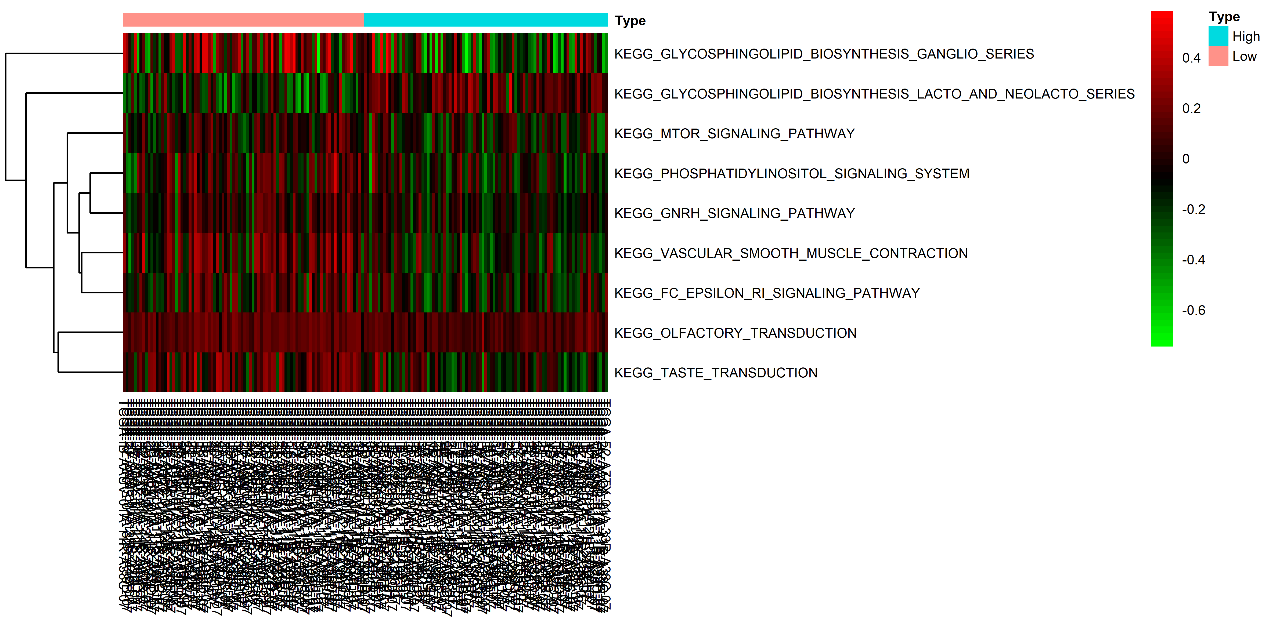


**Supplementary Figure 2** GSVA analysis between High-risk group and Low-risk groups.
